# Presence of gastrointestinal symptoms in IgA nephropathy: a cross-sectional study

**DOI:** 10.1186/s12882-022-03019-8

**Published:** 2022-12-08

**Authors:** Jussi T. Pohjonen, Katri M. Kaukinen, Martti J. Metso, Rakel KK. Nurmi, Heini SA. Huhtala, Ilkka H. Pörsti, Jukka T. Mustonen, Satu M. Mäkelä

**Affiliations:** 1 Celiac Disease Research Center, Faculty of Medicine and Health Technology, Tampere University, FIN-33014 Tampere, Finland; 2 Department of Internal Medicine, Tampere University Hospital, Tampere, Finland; 3 Faculty of Social Sciences, Tampere University, Tampere, Finland; 4 Faculty of Medicine and Health Technology, Tampere University, Tampere, Finland

**Keywords:** Chronic kidney disease (CKD), Gastrointestinal Symptom Rating Scale (GSRS), IgA nephropathy (IgAN), Psychological General Well-Being Index (PGWB)

## Abstract

**Background:**

Gastrointestinal (GI) symptoms are common in end-stage kidney disease. Mounting evidence indicates that the intestine plays an important role in the pathogenesis of IgA nephropathy (IgAN). However, no studies have addressed the obvious question; do IgAN patients suffer from GI symptoms?

**Methods:**

Presence of GI symptoms and health-related quality of life were evaluated using the validated Gastrointestinal Symptom Rating Scale (GSRS) and Psychological General Well-Being (PGWB) questionnaires in 104 patients with kidney biopsy-verified IgAN and in 147 healthy controls. A person was regarded to experience ‘increased GI symptoms’ if the GSRS score exceeded plus 1 standard deviation of the mean of the corresponding score in the healthy controls.

**Results:**

According to the GSRS total score, the IgAN patients had more GI symptoms than the healthy controls (2.0 vs. 1.7, *p* < 0.001). Female IgAN patients had higher GSRS total score than male patients (2.2 vs. 1.7, *p* = 0.001). More IgAN patients with preserved kidney function (eGFR > 60ml/min/1.73m^2^) suffered from increased symptoms of diarrhoea (76 vs. 25%, *p* = 0.028), constipation (81 vs. 19%, *p* = 0.046) and reflux (85 vs. 15%, *p* = 0.004) than did IgAN patients with reduced kidney function (eGFR < 60ml/min/1.73m^2^).

**Conclusions:**

IgAN patients and especially female IgAN patients experienced more GI symptoms than healthy controls. More prevalent GI symptoms were already observed before kidney function was clearly reduced. Systematic enquiry of GI symptoms might increase the standard of care among IgAN patients. Moreover, GI symptoms may provide clues for future studies that examine the pathophysiology of IgAN.

## Introduction

IgA nephropathy (IgAN) is the most common primary glomerulonephritis globally and a notable cause of chronic kidney disease (CKD) and kidney failure [1]. Although the pathogenetic mechanisms of IgAN have not been fully determined, there is mounting evidence that abnormal mucosal immune responses, especially in the intestine, play a role in the disease process [2, 3].

An increased intestinal permeability is present in IgAN [4]. Subclinical small bowel mucosal inflammation has been demonstrated in IgAN patients [5, 6]. The finding of an abundance of intestinal intraepithelial T lymphocytes in IgAN has since been replicated [7]. Associations between IgAN and coeliac disease (CD) are convincing [8–12]. Kidney involvement in inflammatory bowel disease (IBD) is well documented [12–16]. Furthermore, therapy with enteric budesonide targeted at the intestine has reportedly reduced the amount of albuminuria in IgAN [17].

Gastrointestinal (GI) symptoms are common in patients with renal insufficiency [18–20]. The prevalence of GI symptoms has mostly been studied in patients with end-stage kidney disease (ESKD) or after kidney transplantation [21–27]. Only few studies have examined the presence of GI symptoms in patients with less advanced CKD [28, 29].

The aim of this study was to evaluate the presence of GI symptoms in IgAN patients with no diagnosed enteropathies and not progressed to ESKD, and to compare the results to those of healthy controls. Health-related quality of life was surveyed simultaneously. We also aimed to identify the predictors of the assumed GI complaints in IgAN.

## Materials and methods

### Design and study population

A single-centre cross-sectional study was carried out at the Tampere University Hospital (TAUH) and Tampere University, Finland. The TAUH district is an area with a high standard of living and a population of more than 500.000, nearly totally of Caucasian origin. About one hundred kidney biopsies are taken in TAUH annually due to clinical indications [30]. A total of 533 patients with biopsy-proven IgAN diagnosed between 1980 to 2018 were included. The definition of IgAN was based on clinical details and on the presence of glomerular IgA as the sole or predominant immunofluorescence finding [31].

The clinical histories of the 533 IgAN patients were collected from the medical records. Based on the following predefined exclusion criteria, 327 patients were excluded: death (*n* = 51), progression to ESKD (defined as estimated glomerular filtration rate (eGFR) < 15/ml/min/1.73m^2^, initiation of maintenance dialysis or kidney transplantation, *n* = 64), age under 18 or over 80 at recruitment (*n* = 78), known chronic enteropathy (CD or IBD, *n* = 16) [12], moving to another hospital district (*n* = 79), or other obvious reason for exclusion (major GI surgery performed, missing contact information, short life-expectancy for any reason, or a labile mental disorder, *n* = 39). Study questionnaires were posted to the remaining 206 patients in August 2019. The patients were requested to return the questionnaires and the signed informed consent forms within one month. For those who did not return the forms, we verified the contact information and posted the forms in a similar manner once again.

The healthy controls were selected from a group originally comprising 160 people, who had participated in our earlier study [32]. As we restricted them to the same age (18 to 80) as the IgAN patients, the remaining number of available healthy controls was 147. The group was used for the comparison of gastrointestinal symptoms and quality of life. These subjects had no known intestinal diseases at the time of participation, nor did they have first-degree relatives with CD. No laboratory testing had been performed, thus no information, for example on actual kidney function, was available.

### Study questionnaires

For the systematic evaluation of current GI symptoms, all participants completed the self-administered, structured, and well-validated Gastrointestinal Symptom Rating Scale (GSRS) questionnaire [33–35]. The questionnaire evaluates five sub-dimensions of gastrointestinal symptoms: indigestion, diarrhoea, abdominal pain, reflux, and constipation. It comprises altogether 15 separate items. The scoring goes from 1 to 7 points, where 1 point signifies no symptoms, and 7 points signifies the most severe symptoms. The values for each of the five sub-dimension scores were calculated as a mean of the respective items and the total GSRS score as a mean of all 15 items. A person was deemed to suffer from ‘increased GI symptoms’ if the total GSRS score exceeded plus 1 standard deviation (SD) of the mean of the total score in the healthy controls [32]. The same principle was applied to the GSRS sub-scores: ‘increased symptoms’ were taken to be present if an individual’s score exceeded plus 1 SD of the mean of the corresponding sub-score in the healthy controls.

All participants also completed the self-administered, validated questionnaire to measure health-related quality of life, the Psychological General Well-Being (PGWB) questionnaire [36–38]. The survey consists of 22 separate items covering six different sub-dimensions: anxiety, depression, wellbeing, self-control, general health, and vitality. The scoring goes from 1 to 6 points, higher score indicating better quality of life. The sub-dimension scores are calculated as a sum of the items in each sub-dimension and the total PGWB score as a sum of all 22 items.

For the IgAN patients, in addition, information about co-morbidities, and current height and weight was elicited. Tobacco and alcohol consumption were evaluated with standardized tools: The Fagerström Test for Nicotine Dependence and the Alcohol Use Disorders Identification Test (AUDIT-C) questionnaire respectively [39, 40].

### Clinical data

The clinical data for IgAN patients was collected retrospectively from the medical records between 2019 and 2020. We collected data on medications used during the preceding year (antibiotics as courses of treatment), information on possible abdominal surgery and visits and treatments at the nephrological outpatient clinic or ward. Follow-up at the nephrological unit was deemed active if a patient had made one visit during the preceding year and a control visit had been planned. Laboratory test results regarding kidney function were collected for no later than one year preceding the study recruitment.

Kidney function was estimated using the Chronic Kidney Disease Epidemiology Collaboration (CKD-EPI) creatinine equation [41]. eGFR > 60ml/min/1.73m^2^ was regarded as preserved kidney function. Body mass index (BMI) was calculated according to numbers reported in the study questionnaire, by dividing the weight (kilograms) by the square of height (metres).

### Statistical methods

The data are presented as medians and interquartile ranges (IQR) for most of the continuous variables and as percentages for the categorical variables. If a patient failed to answer one or two items in the GSRS or PGWB questionnaires, the missing answer was replaced by the mean value of the other scores for the same subject. If more than two answers were missing, the questionnaire was rejected.

The groups were compared using the Chi-square test, Fisher’s exact test or the Mann–Whitney *U*-test, as appropriate. Binary logistic regression analysis was applied to identify factors for increased GI symptoms. Three covariates were used to avoid overfitting the model. The associations are presented as odds ratios (OR) with 95% confidence intervals (CI).

All tests were two-sided, and *p*-values less than 0.05 were considered statistically significant. All statistical testing was performed using SPSS version 27.0 (IBM SPSS, NY, USA).

### Ethical considerations

The study protocol was approved by the Ethics Committee of the Tampere University Hospital (R18215). All study participants provided written informed consent.

## Results

### Clinical characteristics

Altogether 104 IgAN patients participated in the study. Median age was 55 years, 54% were males and median BMI was 29. Median time from the kidney biopsy was 11 years. Information about current kidney function was available from 82 patients and was preserved (eGFR > 60ml/min/1.73m^2^) in 56% of them. Sixty-three per cent of the patients with preserved kidney function were females.

Female IgAN patients were younger, their BMI was slightly lower, they had better kidney function, and they more often reported concomitant thyroid diseases than male patients. IgAN patients’ detailed characteristics are presented sex-based in Table 1.

**Table 1:** Clinical characteristics of the 104 IgA nephropathy patients. Values are medians (interquartile range) unless otherwise indicated

Patient characteristics	Females	Males	*p*-value
Number of subjects	48	56	
Current age, years	52 (37–58)	59 (51–70)	< 0.001
Current body mass index, kg/m^2^	27 (24–32)	29 (27–33)	0.048
Current smoker, %	19	7	0.135
Risky alcohol use^a^, %	23	29	0.654
Coexisting conditions, %			
Hypertension	40	63	0.030
Diabetes	8	16	0.373
Asthma or chronic obstructive pulmonary disease	17	14	0.790
Sleep apnoea	10	10	1.000
Rheumatic disease	13	7	0.507
Thyroid disease	21	4	0.011
Cancer; active or treated	6	2	0.333
Depression	10	5	0.334
Medication during the year before the study, %			
Blood pressure lowering	69	84	0.101
Lipid lowering	31	43	0.310
Glucose lowering	9	13	0.750
Immunosuppressive	8	4	0.299
Antibiotics	50	43	0.555
Proton pump inhibitor	23	23	1.000
Active follow-up at the nephrological unit, %	21	23	0.816
Time since diagnostic kidney biopsy, years	10 (4–20)	11 (6–20)	0.150
Current kidney function, data available from 41 female and 41 male patients			
Estimated glomerular filtration rate (eGFR), ml/min/1.73m^2^	78 (54–93)	54 (36–68)	< 0.001
eGFR < 60 ml/min/1.73m^2^, %	29	59	0.014

^a^For women 5 or more points, for men 6 or more points in the Alcohol Use Disorders Identification Test (AUDIT−C)

There was a clear female preponderance (72%) among the healthy controls. The median age of the healthy controls was 54 years (42–68), so the groups did not differ in age (*p* = 0.979). Women were younger than men in the healthy controls, too (*p* < 0.001).

### Gastrointestinal symptoms and health-related quality of life

Compared to healthy controls, patients with IgAN reported more GI symptoms as determined by higher GSRS total score (2.0 vs. 1.7, *p <* 0.001). The GSRS sub-scores for diarrhoea, indigestion, reflux, and abdominal pain were also significantly higher among the patients than in controls (Table 2). Female IgAN patients had higher GSRS total score and sub-scores of indigestion, constipation and abdominal pain than male IgAN patients (Table 3). Total GSRS score did not differ between male IgAN patients and male controls (1.7 vs. 1.7, *p* = 0.411), but male IgAN patients had a higher sub-score for diarrhoea (1.7 vs. 1.3, *p* = 0.025). GSRS questionnaires were rejected for two participants among the healthy controls, and for one IgAN patient due to insufficient completion of the questionnaire.

**Table 2:** Comparison of GSRS and PGWB scores (median and interquartile range) between IgA nephropathy (IgAN) patients and healthy controls

	IgAN patients	Healthy controls	*p-*value
GSRS, number of subjects^a^	103	145	
Total score	2.0 (1.5–2.7)	1.7 (1.4–2.2)	< 0.001
* Diarrhoea*	1.7 (1.0-2.7)	1.3 (1.0–2.0)	< 0.001
* Indigestion*	2.5 (2.0-3.3)	2.3 (1.6–2.8)	0.020
* Constipation*	1.7 (1.0-2.3)	1.3 (1.0–2.0)	0.093
* Reflux*	1.5 (1.0-2.5)	1.0 (1.0-1.5)	< 0.001
* Abdominal pain*	1.7 (1.3–2.3)	1.7 (1.0–2.0)	0.006
PGWB, number of subjects^b^	103	142	
Total score	104 (95–113)	109 (101–115)	0.015
* Anxiety*	25 (22–27)	25 (23–27)	0.292
* Depression*	17 (16–18)	17 (16–18)	0.656
* Wellbeing*	17 (15–19)	18 (16–19)	0.585
* Self-control*	16 (15–17)	16 (14–17)	0.243
* General health*	13 (10–14)	15 (13–16)	< 0.001
* Vitality*	17 (15–19)	19 (17–21)	< 0.001

*GSRS* Gastrointestinal Symptom Rating Scale, *PGWB* Psychological General Well−being Index

^a^GSRS rejected for two healthy controls and one IgAN patient due to incomplete questionnaire

^b^PGWB disqualified for five healthy controls and one IgAN patient due to incomplete questionnaire

**Table 3:** Sex-based comparison of GSRS and PGWB scores (median and interquartile range) in IgA nephropathy patients

	Female	Male	*p*-value
GSRS, number of subjects^a^	47	56	
Total score	2.2 (1.8–2.9)	1.7 (1.5–2.3)	0.001
* Diarrhoea*	1.7 (1.0-3.7)	1.7 (1.3–2.3)	0.767
* Indigestion*	3.0 (2.3–3.5)	2.3 (1.8-3.0)	0.005
* Constipation*	2.3 (1.3–3.7)	1.3 (1.0–2.0)	< 0.001
* Reflux*	1.8 (1.0–3.0)	1.5 (1.0–2.0)	0.178
* Abdominal pain*	2.2 (1.7–2.7)	1.7 (1.3-2.0)	< 0.001
PGWB, number of subjects^b^	48	55	
Total score	103 (90–112)	105 (98–114)	0.217
* Anxiety*	25 (20–27)	25 (22–27)	0.145
* Depression*	17 (15–18)	17 (16–18)	0.261
* Wellbeing*	18 (14–19)	17 (16–19)	0.576
* Self-control*	16 (14–17)	16 (15–17)	0.218
* General health*	13 (10–15)	13 (11–14)	0.223
* Vitality*	17 (15–20)	17 (16–19)	0.632

*GSRS *Gastrointestinal Symptom Rating Scale, *PGWB* Psychological General Well−Being Index

^a^GSRS rejected for one IgAN patient due to incomplete questionnaire

^b^PGWB disqualified for one IgAN patient due to incomplete questionnaire

PGWB total score as well as general health and vitality sub-scores were significantly inferior in patients with IgAN than those in healthy controls (Table 2). PGWB scores did not differ between female and male IgAN patients (Table 3). PGWB questionnaires of five participants among the healthy controls and one IgAN patient were rejected due to incomplete response to the questionnaire.

**Fig. 1:**
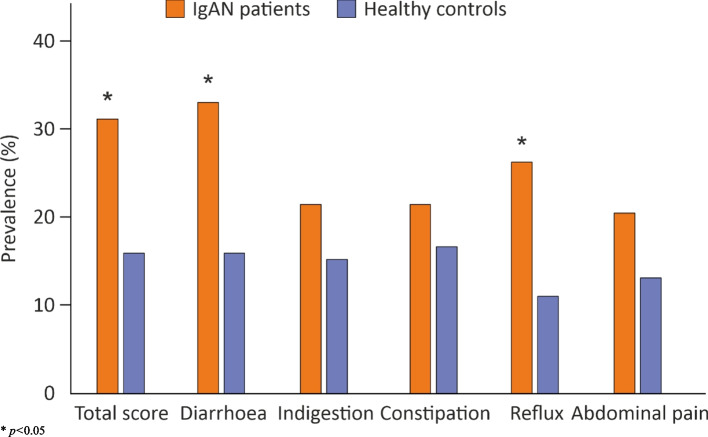
Prevalence of subjects with increased gastrointestinal symptoms in the study groups

**Fig. 2:**
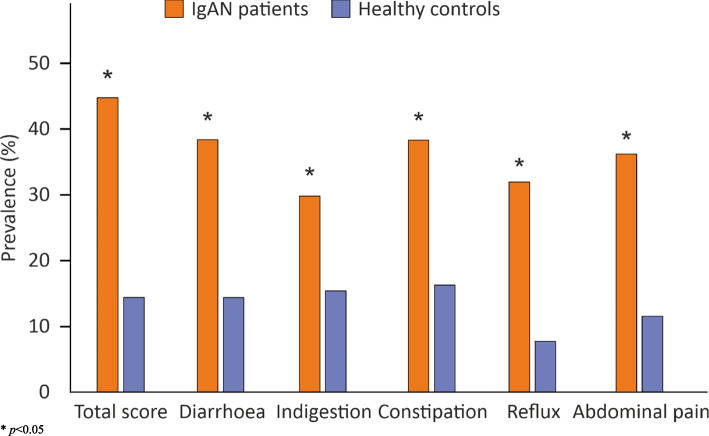
Prevalence of subjects with increased gastrointestinal symptoms in females

Prevalence of increased GI symptoms was more common in IgAN patients than among healthy controls (Fig. 1) and significantly more so when female IgAN patients were compared with healthy women (Fig. 2). No differences were observed in the prevalence of increased GI symptoms between male IgAN patients and healthy males (data not shown). IgAN patients with preserved kidney function reported increased GI symptoms more often than IgAN patients with reduced kidney function in the GSRS sub-scores for diarrhoea (76 vs. 25%, *p* = 0.028), constipation (81 vs. 19%, *p* = 0.046) and reflux (85 vs. 15%, *p* = 0.004). In a multivariable logistic regression analysis of risk factors for increased GI symptoms among IgAN patients, female sex was positively and PGWB total score negatively associated with the higher points in the GSRS total score (Table 4).

**Table 4:** Univariate and multivariable logistic regression analyses of risk factors for increased gastrointestinal symptoms among 103 IgA nephropathy patients

		Univariate				Multivariable	
	OR	95% CI	*p*-value	OR		95% CI	*p*-value
Female sex	3.30	1.38–7.93	0.007	2.80		1.08–7.29	0.035
PGWB total score	0.96	0.93–0.99	0.008	0.96		0.93–1.00	0.026
Age	0.98	0.95–1.01	0.202	1.00		0.97–1.03	0.958
Antibiotic courses	2.25	0.96–5.26	0.063				
Smoking	3.03	0.93–9.91	0.066				
Risky alcohol use^a^	2.24	0.90–5.59	0.084				
Thyroid disease	3.05	0.86–10.85	0.086				
PPI medication	1.85	0.72–4.78	0.204				
eGFR > 60ml/min/1.73m^2^	1.80	0.67–4.87	0.247				
Body mass index	0.97	0.90–1.04	0.359				

*PGWB *Psychological General Well−being Index, *eGFR* estimated glomerular filtration rate, *PPI* proton pump inhibitor

^a^For women 5 or more points, for men 6 or more points in the Alcohol Use Disorders Identification Test (AUDIT−C)

## Discussion

IgAN patients had higher prevalence of GI symptoms than did healthy subjects, and especially female patients with IgAN were more symptomatic than males. An interesting finding was female IgAN patients experiencing increased GI symptoms more often compared to healthy women. Even though male IgAN patients had the same GSRS total score as healthy males, they had higher scores regarding diarrhoea. Interestingly, IgAN patients with preserved kidney function reported more often increased symptoms of diarrhoea, constipation, and reflux than patients with reduced kidney function. IgAN patients experienced poorer health-related quality of life than healthy controls, especially regarding general health and vitality. Furthermore, poorer quality of life associated with increased GI symptoms.

GI symptoms have not previously been evaluated in such a well-defined kidney disease population as in the present study. The fact that diagnosed intestinal diseases were excluded from the current study diminishes the possibility of the findings being biased by the presence of concomitant IBD or CD. In an earlier study where variable diseases had led to CKD stage 4 but dialysis was not yet required (eGFR < 25 ml/min/1.73m^2^), the median of the GSRS total score was 1.84 and the median for the sub-scores were as follows: diarrhoea 1.67, indigestion 2.12, constipation 1.67, reflux 1.00 and abdominal pain 1.50 [21]. In the present study, all sub-scores, and thus also the total score, were at least as high among the IgAN patients. The GSRS total score of 2.0 (1.5–2.7) in IgAN patients in our study was close to the score of the peritoneal dialysis (PD) patients in the aforementioned study (2.07, 1.48–3.08) [21]. PD patients are well known to suffer from excess of GI symptoms [25, 26, 42].

Why did female IgAN patients suffer more often from GI symptoms than male IgAN patients? In general, irritable bowel syndrome (IBS) is more common in women than in men [43]. IBS was also more common among Turkish female than male dialysis patients [44]. Yet, no differences were found in the presence of GI symptoms between sexes in two studies evaluating ESKD patients [21, 42]. Among Australian kidney transplant patients, women again experienced more GI symptoms [27]. Thirty-nine percent of those patients had glomerulonephritis as the primary kidney disease. The sex disparity was speculated to be explained by e.g. hormonal levels, composition of gastrointestinal microbiota, and the finding of women reporting more GI symptoms to health care professionals when compared with men [27]. One explanation could be abdominal discomfort and pain related to the menstrual cycle in fertile-aged women. Still, despite any of the reasons speculated above, female controls in the current study experienced significantly less GI symptoms compared to female IgAN patients.

The present study was unique in focusing on patients with fairly well preserved kidney function; over 50% of the patients had eGFR above 60 ml/min/1.73m^2^. Conventionally, the presence of GI symptoms has been studied in patients who have progressed to ESKD [25, 42]. To the best of our knowledge, only one study has so far evaluated the presence of GI symptoms with the GSRS questionnaire in CKD patients not on dialysis and before transplantation [21]. The other two studies that evaluated the presence of GI symptoms in patients who had not progressed to ESKD used a self-administered patient symptom form, which elicited GI symptoms originally presented in the Modification of Diet in Renal Disease (MDRD) study [28, 29] ‘Abdominal bloating or gas’ were among the most commonly reported symptoms in the MDRD study [28]. Moreover, the GI symptoms were reported to emerge long before ESKD, but still only after eGFR had fallen below 45 ml/min/1.73m^2 ^[28]. In the latter study, GI symptoms became more common as kidney function declined, the one exception being ‘abdominal bloating or gas’, which was equally common and frequently experienced with preserved kidney function [29]. So, why did IgAN patients with better kidney function report increased symptoms of diarrhoea, constipation, and reflux more often than those with reduced kidney function in our study? Etiology of GI symptoms is multifactorial, e.g. higher age diminishes the prevalence of IBS symptoms whereas increased IP and mucosal inflammation likely activate visceral pain in some IBS patients [43, 45]. If GI tract plays a role in the pathogenesis of IgAN, the possible mechanisms might differ between the early and the advanced stages of the disease.

A well-defined and -sized group of patients with a definite diagnosis of IgAN is the strength of the current study. The male/female rate was similar as has been previously reported in IgAN and other glomerular diseases [30]. Exclusion of previously diagnosed intestinal diseases enabled us to focus on the study hypothesis of IgAN patients experiencing GI symptoms without clinically evident enteropathies. Information about kidney function was available in most cases and was most often regarded as normal, making our study population unique in abandoning the conventional idea of GI symptoms becoming prevalent first in advanced CKD.

Our study has some limitations. There was no control group with primary glomerulonephritides, which leaves it uncertain whether the patients experienced an excess of GI symptoms due to kidney disease in general or were the symptoms related with IgAN. The study was carried out in one centre, thus weakening the generalisability of the results. The two study groups had answered the questionnaires years apart. Yet, the results of the healthy controls were consistent with the controls in previous studies [21, 34]. The kidney function was regarded as current, despite the laboratory tests had in many cases been taken months before the study participation, and the information was not available for one fifth of the IgAN patients at all. Taking the most often slowly progressive or stable nature of IgAN into account, it’s unlikely that a significant proportion of the study patients would have had a rapidly progressive disease. Symptomatic patients might have participated more eagerly than asymptomatic patients. This too seems unlikely, as more than one half of the participating IgAN patients were men and their GSRS total score was comparable to that in the healthy controls. Female IgAN patients reported more often thyroid diseases than males, which might explain some of the GI symptoms experienced by females. Thyroid dysfunction in general is more prevalent in women compared to men [46]. Usually GI symptoms in thyroid diseases resolve with treatment [47].

## Conclusions

IgAN patients and especially female IgAN patients suffered from excess GI symptoms even though kidney function was well preserved, and no enteropathies had been diagnosed. Male IgAN patients had also higher scores regarding the presence of diarrhoea than male controls. More prevalent GI symptoms were associated with poorer quality of life, a finding consistent with previous studies [21, 23, 27]. The present findings suggest that routine eliciting of IgAN patients’ GI symptoms would be an appropriate clinical practice. Even though GI symptoms have multifactorial explanations and are not related to IgAN only, perhaps symptoms like presence of loose stools could guide future studies in revealing the complex pathophysiology of IgAN.

## Data Availability

The datasets used and analysed during the current study are available from the corresponding author on reasonable request.

## Abbreviations

BMI: body mass index
CD: coeliac disease
CI: confidence interval
CKD: chronic kidney disease
eGFR: estimated glomerular filtration rate
ESKD: end-stage kidney disease
GI: gastrointestinal
GSRS: Gastrointestinal Symptom Rating Scale
IBD: inflammatory bowel disease
IBS: irritable bowel syndrome
IgAN: IgA nephropathy
IP: intestinal permeability
IQR: interquartile range
PD: peritoneal dialysis
PGWB: Psychological General Well-Being Index
